# High-risk human papillomavirus status and prognosis in invasive cervical cancer: A nationwide cohort study

**DOI:** 10.1371/journal.pmed.1002666

**Published:** 2018-10-01

**Authors:** Jiayao Lei, Alexander Ploner, Camilla Lagheden, Carina Eklund, Sara Nordqvist Kleppe, Bengt Andrae, K. Miriam Elfström, Joakim Dillner, Pär Sparén, Karin Sundström

**Affiliations:** 1 Department of Medical Epidemiology and Biostatistics, Karolinska Institutet, Stockholm, Sweden; 2 Department of Laboratory Medicine, Karolinska Institutet, Stockholm, Sweden; 3 Center for Research and Development, Uppsala University, Region Gävleborg, Sweden; 4 Regional Cancer Center Stockholm–Gotland, Stockholm, Sweden; 5 Karolinska University Laboratory, Karolinska University Hospital, Stockholm, Sweden; Vanderbilt University School of Medicine, UNITED STATES

## Abstract

**Background:**

High-risk human papillomavirus (hrHPV) infection is established as the major cause of invasive cervical cancer (ICC). However, whether hrHPV status in the tumor is associated with subsequent prognosis of ICC is controversial. We aim to evaluate the association between tumor hrHPV status and ICC prognosis using national registers and comprehensive human papillomavirus (HPV) genotyping.

**Methods and findings:**

In this nationwide population-based cohort study, we identified all ICC diagnosed in Sweden during the years 2002–2011 (4,254 confirmed cases), requested all archival formalin-fixed paraffin-embedded blocks, and performed HPV genotyping. Twenty out of 25 pathology biobanks agreed to the study, yielding a total of 2,845 confirmed cases with valid HPV results. Cases were prospectively followed up from date of cancer diagnosis to 31 December 2015, migration from Sweden, or death, whichever occurred first. The main exposure was tumor hrHPV status classified as hrHPV-positive and hrHPV-negative. The primary outcome was all-cause mortality by 31 December 2015. Five-year relative survival ratios (RSRs) were calculated, and excess hazard ratios (EHRs) with 95% confidence intervals (CIs) were estimated using Poisson regression, adjusting for education, time since cancer diagnosis, and clinical factors including age at cancer diagnosis and International Federation of Gynecology and Obstetrics (FIGO) stage. Of the 2,845 included cases, hrHPV was detected in 2,293 (80.6%), and we observed 1,131 (39.8%) deaths during an average of 6.2 years follow-up. The majority of ICC cases were diagnosed at age 30–59 years (57.5%) and classified as stage IB (40.7%). hrHPV positivity was significantly associated with screen-detected tumors, young age, high education level, and early stage at diagnosis (*p <* 0.001). The 5-year RSR compared to the general female population was 0.74 (95% CI 0.72–0.76) for hrHPV-positive cases and 0.54 (95% CI 0.50–0.59) for hrHPV-negative cases, yielding a crude EHR of 0.45 (95% CI 0.38–0.52) and an adjusted EHR of 0.61 (95% CI 0.52–0.71). Risk of all-cause mortality as measured by EHR was consistently and statistically significantly lower for cases with hrHPV-positive tumors for each age group above 29 years and each FIGO stage above IA. The difference in prognosis by hrHPV status was highly robust, regardless of the clinical, histological, and educational characteristics of the cases. The main limitation was that, except for education, we were not able to adjust for lifestyle factors or other unmeasured confounders.

**Conclusions:**

In this study, women with hrHPV-positive cervical tumors had a substantially better prognosis than women with hrHPV-negative tumors. hrHPV appears to be a biomarker for better prognosis in cervical cancer independent of age, FIGO stage, and histological type, extending information from already established prognostic factors. The underlying biological mechanisms relating lack of detectable tumor hrHPV to considerably worse prognosis are not known and should be further investigated.

## Abstract 2

In a study of Swedish medical records and tissue archives, Jiayao Lei and colleagues examine the link between hrHPV positivity in cervical tumors and prognosis.

## Author summary

**Why was this study done?:**

- Studies on tumor human papillomavirus (HPV) status and prognosis of invasive cervical cancer (ICC) have been continually published since the 1990s; however, there have been substantial variations regarding sample size, HPV detection methods in laboratories, and, to some extent, statistical methods.
- Very few studies have comprehensively tested a wide spectrum of both high-risk HPV (hrHPV) and non-high-risk HPV types in cervical tumors when evaluating the association between tumor HPV status and cervical cancer prognosis.
- Controversies remain on the association between tumor hrHPV status and ICC prognosis.

**What did the researchers do and find?:**

- We performed a nationwide population-based study of ICC cases diagnosed in Sweden during the years 2002–2011 in which we comprehensively tested for 13 hrHPV types and 24 HPV types not established as oncogenic.
- The association between hrHPV tumor status and ICC prognosis was assessed, and we found that women with hrHPV-positive tumors had 39% lower excess mortality than women with hrHPV-negative tumors, irrespective of other standard factors for ICC prognosis including patient age, cancer stage, and tumor histological type.

**What do these findings mean?:**

- hrHPV status in invasive tumor tissue appears to be a prognostic biomarker of ICC and could add value to the factors currently considered in clinical decision-making.
- The underlying biological mechanisms relating lack of detectable tumor hrHPV to considerably worse prognosis are not known and should be further investigated.

## Introduction

Cervical cancer is a major cause of morbidity and mortality in women worldwide. The role of high-risk human papillomavirus (hrHPV) in the development of invasive cervical cancer (ICC) is well established [1]. Persistent infection with hrHPV in the cervical epithelium, especially types HPV16 and HPV18, is known to be associated with higher probability of progression to cervical intraepithelial lesion grade 3 (CIN3) and ICC compared to being negative for such infection [2]. However, once a cancer has occurred, the extent to which hrHPV status in the actual invasive tumor tissue may be related to prognosis of ICC has been found to be variable between studies [3–9]. This is despite the fact that for oropharyngeal cancer, another tumor etiologically linked to hrHPV, there is consensus that presence of hrHPV in the tumor marks better prognosis [10,11].

The disparities of existing findings on cervical cancer prognosis may be explained by variation in design or assay use or the limited size of the study population. The subject is of importance as a straightforward prognostic biomarker could be considered for clinical use. Large and stringently designed studies are needed to answer the question of whether hrHPV status is related to prognosis. To this end, we conducted a population-based cohort study considering all ICC cases occurring in Sweden during the years 2002–2011, using comprehensive survival and human papillomavirus (HPV) genotyping data and providing a large-scale population-based evaluation of the association between tumor hrHPV status and ICC prognosis.

## Methods

### Study population

**Fig 1:**
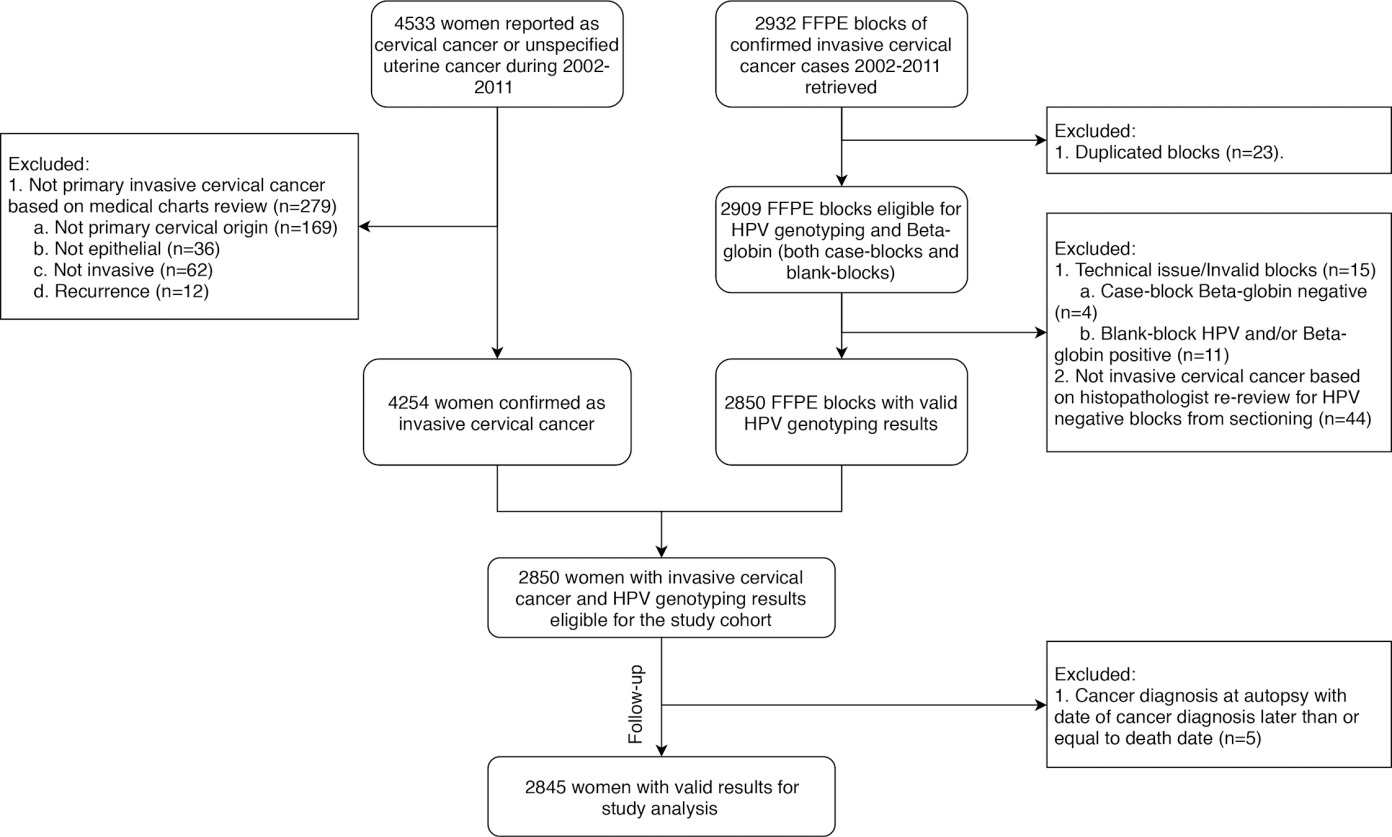
Flow chart of study population. FFPE, formalin-fixed paraffin-embedded; HPV, human papillomavirus.

Records for a total of 4,533 women (Fig 1) diagnosed with cervical cancer or unspecified uterine cancer in Sweden between 1 January 2002 and 31 December 2011 were retrieved from the Swedish Cancer Registry, which includes virtually complete information on all cancer cases diagnosed in Sweden since 1958 [12]. A senior gynecologist (BA) reviewed the medical charts of the 4,533 cases. The 279 cases not confirmed as primary invasive epithelial cancers of cervical origin were excluded, leaving 4,254 confirmed cases. From all confirmed cases, archived formalin-fixed paraffin-embedded (FFPE) blocks obtained from the primary invasive epithelial cervical tumor were requested from the archives of the diagnosing pathology laboratories. Among the 25 pathology biobanks in Sweden, 4 biobanks declined to provide blocks for sectioning and 1 biobank was excluded because of long delays in the approval process. In all, 2,932 blocks were collected. When there was more than 1 diagnostic block, the blocks were evaluated for the proportion of tumor versus healthy tissue and only the block with the highest proportion of tumor tissue was kept, resulting in exclusion of 23 duplicate blocks.

All eligible blocks (*n* = 2,909) were then subjected to HPV genotyping. Blocks with invalid results, including case-blocks showing β-globin-negative results (*n* = 4), and cases with corresponding blank-blocks showing HPV-positive and/or β-globin-positive results (*n* = 11) were excluded. All blocks from primary cervical cancer with HPV-negative results were re-reviewed by our pathologist (W. Ryd) to confirm or reject ICC diagnosis. Blocks identified as not containing any ICC tumor tissues (*n* = 44) were excluded. In the end, a total of 2,850 confirmed cases with valid HPV genotyping results were eligible for our cohort.

Our main exposure was tumor hrHPV status. HrHPV-positive cases included cases that were positive for only hrHPV or both hrHPV and low-risk HPV, while hrHPV-negative cases included both cases without any detectable HPV and cases positive for only low-risk HPV. Women were prospectively followed up from date of cancer diagnosis to 31 December 2015, migration from Sweden, or death, whichever occurred first. Information on migration and death was retrieved from the Total Population Register [13] using the individually unique national personal identification number for all Swedish residents. Women with cancer diagnosis upon autopsy were excluded. A total of 2,845 women were thus eligible for our analyses. All analyses were carried out blinded to study endpoint.

### HPV genotyping and validation

All FFPE blocks were extracted and tested in parallel with β-globin real-time polymerase chain reaction (PCR) and HPV genotyping using general primers (MGP)-PCR targeting the L1 region [14], followed by typing with Luminex for 13 high-risk types (16, 18, 31, 33, 35, 39, 45, 51, 52, 56, 58, 59, 68; 12 of these types are established as class I carcinogens and HPV68 is classified as a probable carcinogen) and 24 HPV types not established as oncogenic (i.e. low-risk) (6, 11, 26, 30, 40, 42, 43, 53, 54, 61, 66, 67, 69, 70, 73, 74, 81, 82, 83, 86, 87, 89, 90, 91) [15,16]. A blank-block containing only paraffin was sectioned and analyzed in between each case block as a control for contamination. The blank-blocks had to be negative in all tests to confirm lack of contamination, and the case-block had to be positive for β-globin to confirm the existence of human tissue.

Real-time E7 and E6 PCR tests for HPV16 and HPV18 were further performed for all tumors that were HPV-negative in the L1-directed PCR [17], as loss of the L1 region could occur in tumors, whereas the oncogenic E7 and E6 regions are more likely to be retained [18].

### Statistical analysis

We used Pearson’s chi-squared test to compare the distribution of age, International Federation of Gynecology and Obstetrics (FIGO) stage, histological type, mode of detection, education, and hrHPV status for included cases. Age at cancer diagnosis was grouped as <30, 30–44, 45–59, 60–74, and >74 years. FIGO stage was categorized as IA, IB, II, and III+ [19]. Mode of detection was categorized as screen-detected or symptomatic cancer, based on our review of medical charts. Information on education was retrieved from the Swedish Longitudinal Integration Database for Health Insurance and Labour Market Studies (LISA) [20] and classified as low, middle, high, or missing based on the highest education level achieved by time of cancer diagnosis. Tumor grade was available for a subset of tumors; those in which cancer cells obviously presented as low differentiation in histopathological assessment were classified as high grade.

All-cause mortality was modeled as relative survival in relation to the general female population in Sweden with comparable age and during the same calendar period, with time since cancer diagnosis as time scale. We used the life table method [21] to estimate relative survival ratios (RSRs) with 95% confidence intervals (CIs) for cumulative time since diagnosis by 1-year increments, separately for hrHPV-positive and hrHPV-negative cases. We used Poisson regression to estimate crude and adjusted excess hazard ratios (EHRs) with 95% CIs for 5-year survival; this corresponds to an additive hazards model with a baseline hazard based on the general population and an excess hazard component estimated from the cohort at hand ([21], Section 5, Eq 1), with survival censored at 5 years. For all Poisson models, we assumed approximately constant hazard across 1-year time bands and split the follow-up time accordingly. In adjusted models, we included age at cancer diagnosis as a spline term with 5 degrees of freedom, time since cancer diagnosis corresponding to the 1-year time bands, FIGO stage, and education. We did not adjust for treatment type since we found it overlapped substantially with stage. In stratified analyses, we also calculated the 5-year RSRs, crude 5-year EHRs, and adjusted 5-year EHRs with 95% CIs by age group and FIGO stage. In all stratified adjusted models, age at cancer diagnosis was included as a spline term with 3 degrees of freedom. We further selected squamous cell carcinoma (SCC) and adenocarcinoma (AC), the 2 main histological types of ICC, to examine the association of hrHPV status and prognosis by histological type.

### Sensitivity analysis

We compared the clinical and educational characteristics for confirmed ICC cases with and without FFPE blocks retrieved, using Pearson’s chi-squared test. We calculated crude and adjusted 1-year, 3-year, 5-year, and 10-year EHRs by hrHPV status. We applied a stratified adjusted model by mode of detection and a subgroup analysis by utilizing information from tumors classified as high grade (*n* = 221). Moreover, we reclassified hrHPV status taking also HPV16-E7 and HPV18-E6 real-time PCR results into account. For that analysis of relative survival and excess mortality, tumors HPV negative in Luminex but HPV16-E7 and/or HPV18-E6 real-time PCR positive (*n* = 89) were considered hrHPV positive and analyzed together with hrHPV-positive cases. Finally, we linked 2,845 included cases to the Swedish National Cervical Screening Registry (NKCx) for identifying those cases that had HPV testing results prior to cancer diagnosis (*n* = 55).

Analyses were performed in SAS 9.4 (SAS Institute) and Stata 15.1 (StataCorp). *p*-Value *<* 0.05 was considered statistically significant. We present the original analysis protocol and the documentation of revision during the whole analysis process with reasons for changes in S1 Text. This study was approved by the Regional Ethical Review Board in Stockholm, which determined that, due to the population-based nature of the study, informed consent from the study participants was not required (Dnr 2011/1026-31/4; Dnr 02–556; Dnr 2012/1028/32; Dnr 2011/921-32).

## Results

### Determinants of hrHPV status

hrHPV was detected in tumors of 2,293 cases. Compared to hrHPV-negative cases, hrHPV-positive cases were more likely to be diagnosed at younger age, to be diagnosed at an earlier FIGO stage, to be classified as having a screen-detected cancer, and to have a higher education (all *p <* 0.001; Table 1).

**Table 1:** Characteristics of women with a primary invasive cervical cancer diagnosis 2002–2011 in Sweden by tumor hrHPV status.

Characteristic	Tumor hrHPV status		Total	*p*-Value*
	hrHPV+	hrHPV−		
**Number of cases**	2,293	552	2,845	
**Number of deaths, number (%)**	822 (36.3)	309 (56.0)	1,131 (39.8)	
**Total person-years**	14,990.1	2,707.9	17,711.0	
**Mean follow-up years (SE)**	6.5 (0.08)	4.9 (0.18)	6.2 (0.07)	
**Age at cancer diagnosis, number (%)**				<0.001
<30	161 (7.0)	6 (1.1)	167 (5.9)	
30–44	822 (35.8)	111 (20.1)	933 (32.8)	
45–59	580 (25.3)	122 (22.1)	702 (24.7)	
60–74	394 (17.2)	146 (26.4)	540 (19.0)	
>74	336 (14.7)	167 (30.3)	503 (17.7)	
**FIGO stage, number (%)**				<0.001
IA	455 (19.8)	75 (13.6)	530 (18.6)	
IB	972 (42.4)	185 (33.5)	1,157 (40.7)	
II	462 (20.1)	115 (20.8)	577 (20.3)	
III+	404 (17.6)	177 (32.1)	581 (20.4)	
**Histological type, number (%)**				<0.001
Squamous cell carcinoma	1,735 (75.7)	374 (67.8)	2,109 (74.1)	
Adenocarcinoma	410 (17.9)	116 (21.0)	526 (18.5)	
Adenosquamous cell carcinoma	88 (3.8)	31 (5.6)	119 (4.2)	
Other rare carcinomas	60 (2.6)	31 (5.6)	91 (3.2)	
**Mode of detection, number (%)**				<0.001
Symptomatic cancer	1,593 (69.5)	444 (80.4)	2,037 (71.6)	
Screen-detected cancer	700 (30.5)	108 (19.6)	808 (28.4)	
**Education, number (%)**				<0.001
Low	597 (26.0)	215 (38.9)	812 (28.5)	
Middle	1,038 (45.3)	208 (37.7)	1,246 (43.8)	
High	610 (26.6)	113 (20.5)	723 (25.4)	
Missing	48 (2.1)	16 (2.9)	64 (2.2)	

No missing values for age at cancer diagnosis, FIGO stage, histological type, and mode of detection.

*p-Value was determined using chi-squared tests.

FIGO, International Federation of Gynecology and Obstetrics; hrHPV, high-risk human papillomavirus; SE, standard error.

### hrHPV status and prognosis

**Fig 2:**
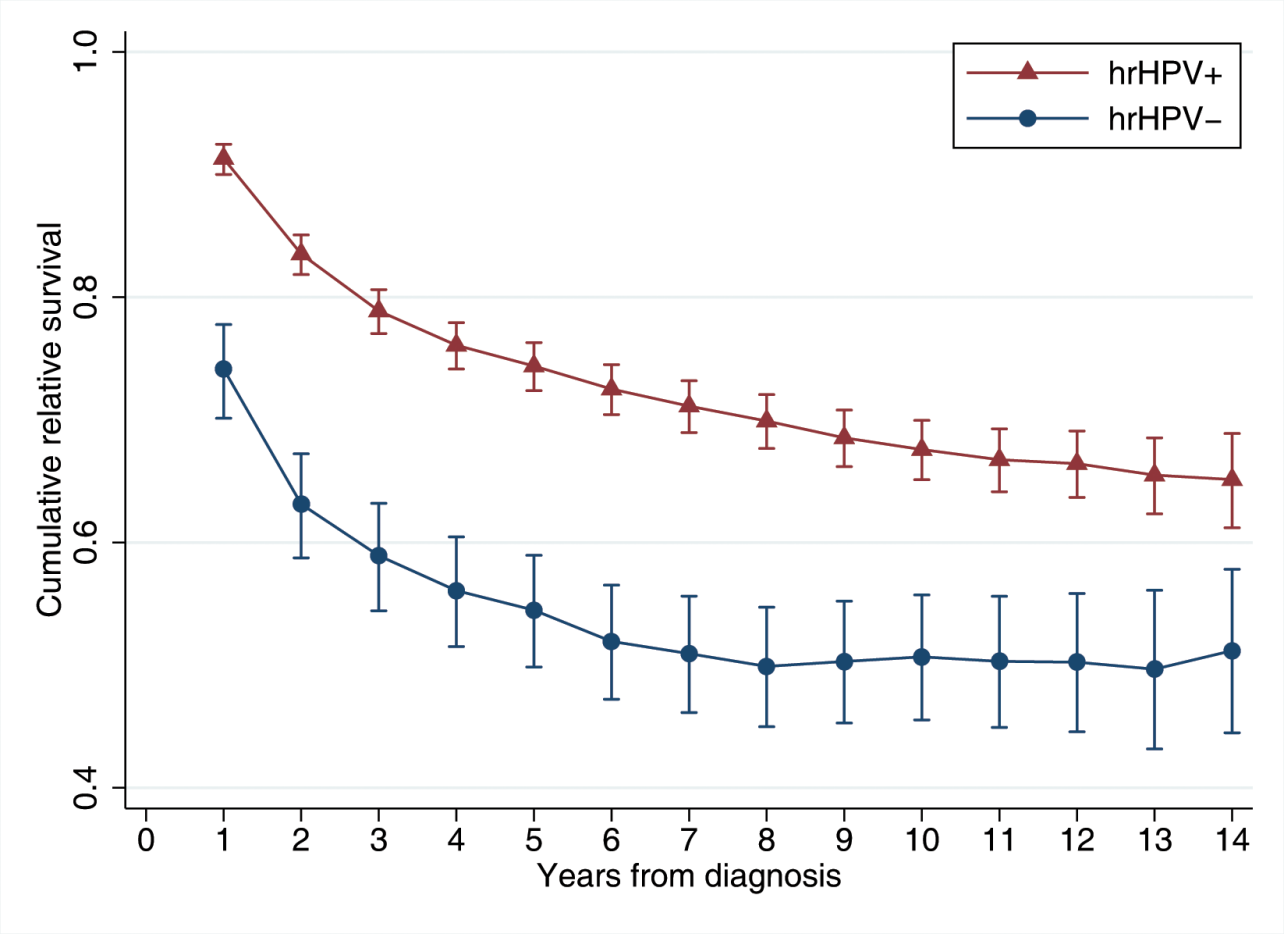
Cumulative relative survival of invasive cervical cancer cases by tumor high-risk human papillomavirus (hrHPV) status. Cumulative relative survival corresponds to the relative survival ratio in relation to the general female population with comparable age and during the same calendar period over the indicated time since diagnosis. *p*-Values of a Wald test between hrHPV-positive cases and hrHPV-negative cases are less than 0.001 across time since cancer diagnosis.

Cumulative relative survival was considerably and constantly higher for hrHPV-positive cases than hrHPV-negative cases throughout the study period (Fig 2). There were 822 deaths (36.3%) observed among hrHPV-positive cases and 309 deaths (56.0%) among hrHPV-negative cases during the study period. The mean follow-up time was 6.5 years for hrHPV-positive cases and 4.9 years for hrHPV-negative cases. Survival decreased sharply for both hrHPV-positive and hrHPV-negative cases within the first 3 years after cancer diagnosis, especially during the first 2 years. In hrHPV-positive cases, the 5-year RSR was 0.74 (95% CI 0.72–0.76) and in hrHPV-negative cases, the RSR was 0.54 (95% CI 0.50–0.59). This yielded a crude EHR of 0.45 (95% CI 0.38–0.52) and an adjusted EHR of 0.61 (95% CI 0.52–0.71), indicating a statistically significant 39% lower excess mortality among hrHPV-positive cases compared to hrHPV-negative cases after adjustment for age at cancer diagnosis, FIGO stage, education, and time since cancer diagnosis (Table 2).

**Table 2:** Five-year relative survival ratios (RSRs) and 5-year excess hazard ratios (EHRs) in relation to tumor high-risk human papillomavirus (hrHPV) status.

hrHPV status	Cases (*n* = 2,845)	Deaths (*n* = 1,131)	5-year RSR (95% CI)	5-year EHR (95% CI)	
				Crude	Adjusted*
hrHPV−	552	309	0.54 (0.50–0.59)	Ref	Ref
hrHPV+	2,293	822	0.74 (0.72–0.76)	0.45 (0.38–0.52)	0.61 (0.52–0.71)

*EHRs were adjusted for age at cancer diagnosis as a spline term with 5 degrees of freedom, time since cancer diagnosis in 1-year bands, International Federation of Gynecology and Obstetrics (FIGO) stage, and education.

**Fig 3:**
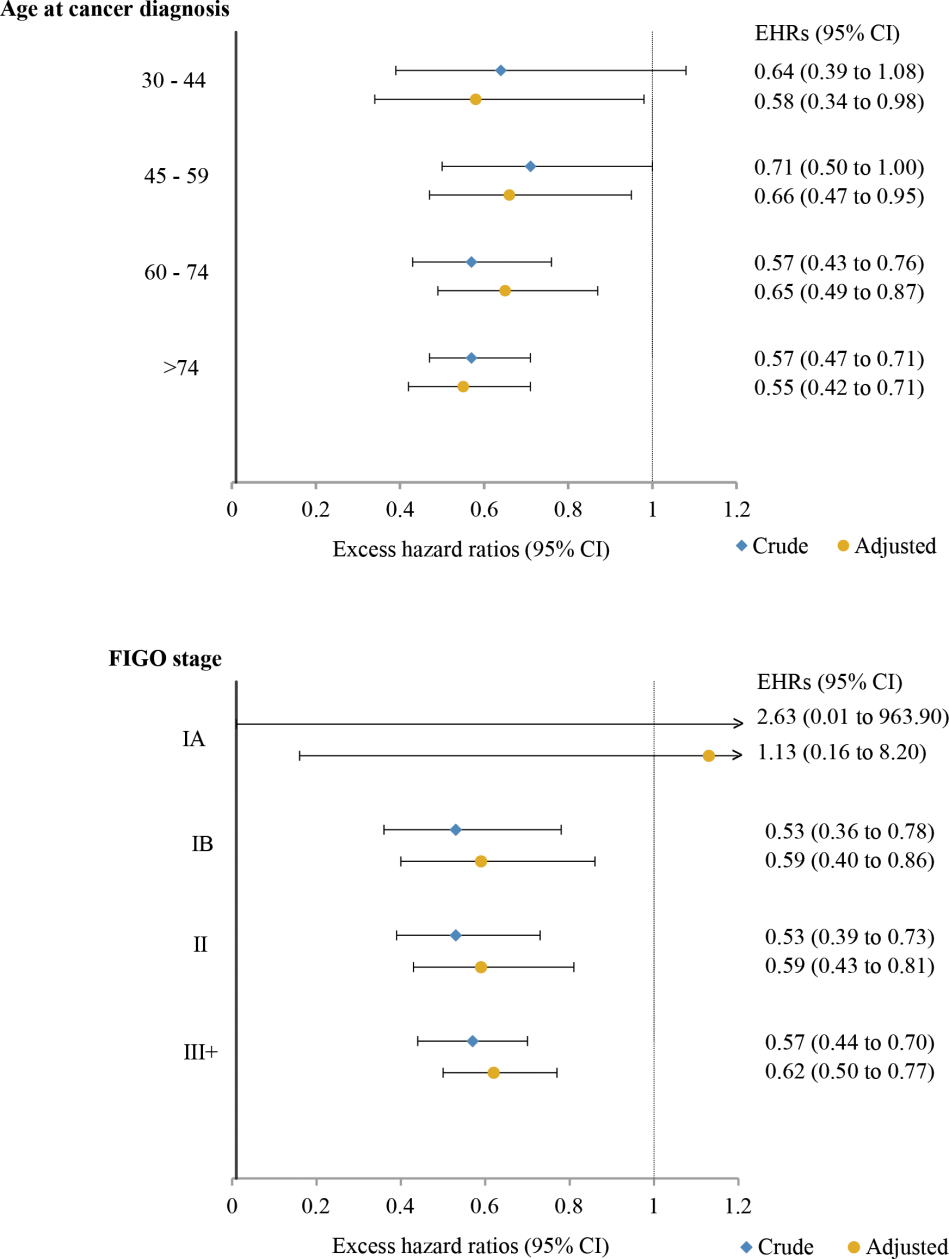
Five-year excess hazard ratios (EHRs) in relation to tumor high-risk human papillomavirus (hrHPV) status by age at cancer diagnosis and International Federation of Gynecology and Obstetrics (FIGO) stage. (A) By age at cancer diagnosis; (B) by FIGO stage. The reference groups are hrHPV-negative cases in the corresponding age group (A) and FIGO stage (B). In (A), EHRs were adjusted for age at cancer diagnosis as a spline term with 3 degrees of freedom, time since cancer diagnosis in 1-year bands, FIGO stage, and education. In (B), EHRs were adjusted for age at cancer diagnosis as a spline term with 3 degrees of freedom, time since cancer diagnosis in 1-year bands, and education. No estimates for women with age at cancer diagnosis under 30 years were included in (A) due to insufficient number of events. Estimates for women diagnosed at stage IA in (B) were truncated for display purposes.

Stratified by age at cancer diagnosis, 5-year RSRs were consistently and statistically significantly higher for hrHPV-positive cases than hrHPV-negative cases in all age groups from age 30 years up (S1 Table). The 5-year age-specific adjusted EHRs for hrHPV-positive cases ranged from 0.55 to 0.66 (Fig 3) compared to hrHPV-negative cases. A similar pattern was seen for cases in each FIGO stage above IA, with adjusted EHRs for hrHPV-positive cases ranging from 0.59 to 0.62 (Fig 3; S2 Table).

### hrHPV status, histological type, and prognosis

Our analysis of hrHPV status and histological type included the 2 main histological types of ICC (2,109 cases of SCC and 526 cases of AC). hrHPV-negative AC was associated with the worst 5-year relative survival (RSR 0.45, 95% CI 0.35–0.55), followed by hrHPV-negative SCC, hrHPV-positive SCC, and hrHPV-positive AC (Table 3). Using hrHPV-negative SCC as reference, hrHPV-positive SCC and hrHPV-positive AC were both related to significantly better prognosis, showing adjusted EHRs of 0.68 (95% CI 0.56–0.82) and 0.61 (95% CI 0.43–0.87), respectively.

**Table 3:** Five-year relative survival ratios (RSRs) and 5-year excess hazard ratios (EHRs) in relation to high-risk human papillomavirus (hrHPV) status in the 2 main histological types of invasive cervical cancer.

Histological type and hrHPV status	Cases (*n* = 2,635)	Deaths (*n* = 1,021)	5-year RSR (95% CI)	5-year EHR (95% CI)	
				Crude	Adjusted*
SCC, hrHPV−	374	190	0.59 (0.53–0.64)	Ref	Ref
SCC, hrHPV+	1,735	665	0.73 (0.70–0.75)	0.56 (0.46–0.68)	0.68 (0.56–0.82)
AC, hrHPV−	116	81	0.45 (0.35–0.55)	1.48 (1.09–2.01)	1.44 (1.07–1.97)
AC, hrHPV+	410	85	0.87 (0.83–0.90)	0.23 (0.17–0.33)	0.61 (0.43–0.87)

Adenosquamous cell carcinoma (*n* = 119) and other rare carcinomas (*n* = 91) are not included in this analysis.

*EHRs were adjusted for age at cancer diagnosis as a spline term with 5 degrees of freedom, time since cancer diagnosis in 1-year bands, International Federation of Gynecology and Obstetrics (FIGO) stage, and education.

AC, adenocarcinoma; SCC, squamous cell carcinoma.

### Sensitivity analyses

Cases with and without FFPE blocks retrieved differed somewhat regarding distribution of FIGO stage and histological type, with fewer stage IA and AC/other rare carcinomas obtained than occurred in the overall material (S3 Table). The 1-year, 3-year, 5-year, and 10-year adjusted EHRs by hrHPV status (S4 Table) showed 51%, 46%, 39%, and 36% lower excess mortality for hrHPV-positive cases compared to hrHPV-negative cases, respectively. In the stratified adjusted model, better prognosis remained for hrHPV-positive cases compared to hrHPV-negative cases irrespective of mode of detection (S5 Table) or high tumor grade (S6 Table). Cases that were only positive for HPV16-E7 or HPV18-E6 but not L1 were had a prognosis similar to that of hrHPV-negative cases (EHR 1.15, 95% CI 0.78–1.69). When we reclassified the cases incorporating HPV16-E7/HPV18-E6 status, the association between hrHPV status and prognosis remained unchanged (S7–S9 Tables).

There were 55 cases that had HPV testing results from liquid-based cytology tests prior to ICC diagnosis (S10 Table); 53/55 (96.4%) pre-diagnostic HPV tests were hrHPV positive. The 2 hrHPV-negative tests before cancer were from (i) a stage II AC diagnosed in 2009 where the HPV test in question was performed 2 years before cancer diagnosis and (ii) an ICC with HPV73 detected by us in the tumor, which was not any of the hrHPV types tested for in screening. Only 4/55 ICC cases that were HPV negative in the tumor had a pre-diagnostic HPV test, out of which 3 had been hrHPV positive.

## Discussion

In this large, nationwide study including 2,845 HPV-genotyped cervical cancer cases occurring over 10 years, we found that women with hrHPV-positive ICC had a statistically significant 39% lower excess mortality compared to women with hrHPV-negative tumors. The substantial survival difference by hrHPV status observed already within 2–3 years after ICC diagnosis illustrates this biomarker’s potential for distinguishing ICC associated with higher mortality. The difference in prognosis was highly robust regardless of the clinical, histological, and educational characteristics of the cases.

Findings from previous studies on tumor hrHPV status and prognosis have not been conclusive [3–9]. Cuschieri et al. [4] found that HPV16- and HPV18-positive ICC cases had improved survival compared to ICC infected by other HPV types. A large study [8] showed that cases with both HPV alpha-7 (which includes HPV18) and alpha-9 (which includes HPV 16) species had better survival compared to cases with only alpha-7 species, and a worse prognosis was also seen for HPV-negative cases. Meanwhile, other studies have reported better prognosis for HPV16 [9] but worse for HPV18 [5]. In addition, findings on overall detectability of HPV have either shown equal [3,6] or worse [4,7,8] prognosis in HPV-negative as compared to HPV-positive cases. Findings on the impact of histological type on prognosis have also been inconsistent [22,23]. Our findings suggest that the proportion of hrHPV-positive and hrHPV-negative cases among the histological types might also explain the discrepancy in prognostic value deriving from the latter variable. Therefore, considering hrHPV status, in addition to evaluating histology and stage, should add substantial prognostic value in the clinical management of ICC.

Although HPV is a necessary but not sufficient cause of ICC, a variable proportion of tumors are reported to be negative for hrHPV [24,25]. We found that the hrHPV status of tumors is strongly dependent on the mode of cancer detection (screening or symptomatic), age at diagnosis, and stage at diagnosis, providing a likely explanation for this variability of the proportion with hrHPV positivity seen in different case series. It is key to note that hrHPV negativity in the tumor does not imply that hrHPV was not involved in the etiology of cancer development, since hrHPV-negative cases may have been infected with hrHPV at an earlier time point before cancer diagnosis. Rather, our findings imply that hrHPV DNA may become undetectable at a late stage of the oncogenic process, as HPV negativity was associated with tumors detected at a late FIGO stage and tumors not detected by screening. Indeed, a similar association between HPV negativity and advanced cancer cases was shown in an earlier study [26]. Also, using the same HPV detection method as in this study, we previously found that 97% of CIN3 or worse cases in the Swedish population were hrHPV positive [27], and similar very high proportions of hrHPV-positive CIN3 cases have been documented by others [28].

The registry linkage analyses of pre-diagnostic HPV tests found that 53/55 (96.4%) HPV tests prior to cancer diagnosis had been hrHPV positive (S10 Table), which was in line with the improved protection against cervical cancer of using primary HPV-based screening in prospective randomized trials [29], implying that screen-detectable cervical cancer precursors are in general hrHPV positive. HPV-based screening was not recommended in Sweden until 2015; thus, the opportunity to examine hrHPV status in screening prior to cancer diagnosis was limited for this cohort of HPV-negative cases. The identification of 3 out of 4 hrHPV-positive pre-diagnostic specimens from women who later had an HPV-negative ICC also supports that in some cases hrHPV may be involved in carcinogenesis, but may be lost in advanced stages of the tumor. Our findings are fully compatible with hrHPV being the cause of cervical cancer, yet equally suggest that loss of hrHPV DNA in the resulting tumor appears to be of clinical importance and should not be ignored.

The inability to detect HPV in tumors might be explained by low viral load of HPV DNA in the tumor tissues [24]; loss of the L1 region, which could occur in some fraction of the cancers [18]; poor quality of samples; undetectable HPV types; or partial loss of HPV because of integration [30]. We therefore used only diagnostic blocks with stringent quality controls, used sensitive laboratory methods capable of identifying 37 HPV genotypes, and in addition included testing for oncogenic E6 and E7 regions of HPV16 and HPV18. It is thus unlikely that we had any substantial proportion of hrHPV negativity deriving from methodological problems. Had hrHPV negativity been caused by methodological problems in our study, misclassifying hrHPV-positive tumors as hrHPV negative, this would have diluted the associations found, and therefore our study would not have been likely to detect such strong associations with the biological behavior of the tumor. Conceivably, some ICC tumors might accumulate genetic changes to the point that continued presence of the hrHPV is no longer necessary to maintain malignancy. Another plausible explanation—which has been proposed to explain the better prognosis of hrHPV-positive oropharyngeal cancer—is that hrHPV-positive cases might be more immunogenic because of continued expression of viral proteins, making tumors more susceptible to control by the immune system [10].

Limitations of our study include that we were not able to collect FFPE blocks for all cases, but the introduction of confounding or selection bias due to this was limited, as it was related to the policy applied by the respective biobank, and thus cannot have been differential on HPV presence in tissue. Furthermore, our sample was shown to be representative of the original case load, where the only difference of note was an over-representation of micro-invasive cancer (stage IA) in excluded cases, attributable to insufficient sample adequacy for re-sectioning based on limited remaining malignant tissue in the archival block. It should be noted, though, that statistical adjustment for FIGO stage or age might not be sufficient to explain prognostic variations within stages or ages; therefore, residual confounding by stage or age might theoretically still exist in our estimates. However, as the magnitude of the association of these factors with hrHPV status was strong and robust under both advanced adjustment and stratification for these factors, we judge that there is little risk that residual confounding could have substantially biased the associations we observed.

Finally, we acknowledge that, except for education, we did not have data on socioeconomic status or lifestyle factors, such as smoking, which may affect ICC prognosis [31]. If unhealthy lifestyle factors were more likely to be associated with hrHPV negativity in the tumor, and adversely related to ICC prognosis, failing to adjust for these factors would result in an underestimation of the excess mortality for women who had a hrHPV-positive tumor. However, we had complete data on education level, which is a good proxy indicator both for overall socioeconomic status [32] and to some extent for smoking status [33]. We thus included education level in our final model to control for potential confounding from different lifestyle habits, and, indeed, our results remained entirely robust. We therefore posit that extensive residual confounding from such factors appears to be unlikely, and that the utility of hrHPV status as a clinical biomarker for prognosis is easier to quantify in practice regardless.

To our knowledge, this is the first, and largest to date, nationwide population-based study of hrHPV status and cervical cancer prognosis. We employed stringently validated methods for ascertainment of hrHPV status and strict clinical and histopathological review for case ascertainment. This study was further augmented with virtually complete and high-quality register data, which enabled us to adjust for potential confounders and minimize loss to follow-up in an unbiased manner. We used relative survival, measuring all-cause mortality in relation to the general female population of the same age and during the same time period. This approach is well suited to adjust for natural mortality in the population, and also eliminates the risk of potential misclassification of causes of death. We also controlled for FIGO stage, which is closely related to mode of cancer detection and alleviates lead time and length time bias in survival studies [23]. Prognosis by hrHPV status remained strongly different also after adjustment for FIGO stage and/or high grade. Importantly, we used established and widely available laboratory methods, suggesting that testing for hrHPV status could be used alongside age and stage at diagnosis in clinical prognostication.

## Conclusions

Since methods of HPV analysis have been substantially improved in recent years, and have also been implemented in cervical screening, hrHPV status in tumors may represent a routinely available biomarker for cervical cancer prognosis of potentially substantial value. We observed that women with hrHPV-positive cervical tumors had a substantially better prognosis than women with hrHPV-negative tumors. We thus posit that hrHPV appears to be a biomarker for ICC prognosis, extending information from the already established prognostic factors age, clinical stage, and histological type. The underlying biological mechanisms relating lack of detectable tumor hrHPV to considerably worse prognosis are not known and should be further investigated.

## Supporting information

S1 Table Five-year relative survival ratios (RSRs) and 5-year excess hazard ratios (EHRs) in relation to high-risk human papillomavirus (hrHPV) status, by age at cancer diagnosis. (DOCX)

S2 Table Five-year relative survival ratios (RSRs) and 5-year excess hazard ratios (EHRs) in relation to high-risk human papillomavirus (hrHPV) status, by FIGO stage. (DOCX)

S3 Table Characteristics of confirmed cases by availability of valid blocks. (DOCX)

S4 Table One-year, 3-year, 5-year, and 10-year excess hazard ratios (EHRs) in relation to high-risk human papillomavirus (hrHPV) status. (DOCX)

S5 Table Five-year relative survival ratios (RSRs) and 5-year excess hazard ratios (EHRs) in relation to high-risk human papillomavirus (hrHPV) status, by mode of detection. (DOCX)

S6 Table Five-year relative survival ratios (RSRs) and 5-year excess hazard ratios (EHRs) in relation to high-risk human papillomavirus (hrHPV) status, in high-grade tumors. (DOCX)

S7 Table Five-year relative survival ratios (RSRs) and 5-year excess hazard ratios (EHRs) in relation to high-risk human papillomavirus (hrHPV) status based on L1 region and HPV16-E7/HPV18-E6. (DOCX)

S8 Table Five-year relative survival ratios (RSRs) and 5-year excess hazard ratios (EHRs) in relation to high-risk human papillomavirus (hrHPV) status based on L1 region and HPV16-E7/HPV18-E6, by age at cancer diagnosis. (DOCX)

S9 Table Five-year relative survival ratios (RSRs) and 5-year excess hazard ratios (EHRs) in relation to high-risk human papillomavirus (hrHPV) status based on L1 region and HPV16-E7/HPV18-E6, by FIGO stage. (DOCX)

S10 Table Tumor human papillomavirus (HPV) status and pre-diagnostic HPV testing results. (DOCX)

S1 Text Analysis protocol. (PDF)

S2 Text STROBE checklist. (DOC)

S3 Text Other supporting tables and figures. (PDF)

## Abbreviations

AC: adenocarcinoma
CI: confidence interval
CIN3: cervical intraepithelial lesion grade 3
EHR: excess hazard ratio
FIGO: International Federation of Gynecology and Obstetrics
FFPE: formalin-fixed paraffin-embedded
HPV: human papillomavirus
hrHPV: high-risk human papillomavirus
ICC: invasive cervical cancer
PCR: polymerase chain reaction
RSR: relative survival ratio
SCC: squamous cell carcinoma
