# Supplementary material for: High-risk human papillomavirus status and prognosis in invasive cervical cancer: A nationwide cohort study
Source: PLoS Med. 2018 Oct 1;15(10):e1002666. doi: 10.1371/journal.pmed.1002666 (PMC6166926; doi:10.1371/journal.pmed.1002666)
Supplement: S1 Table — (DOCX) [file pmed.1002666.s001.docx]

**S1 Table. Five-year relative survival ratios (RSRs) and 5-year excess hazard ratios (EHRs) in relation to high-risk human papillomavirus (hrHPV) status, by age at cancer diagnosis.**

| **Age at cancer diagnosis** | **hrHPV status** | **Deaths**  **(n=1131)** | **5-year RSR**  **(95% CI)** | **5-year crude EHR**  **(95% CI)** | **5-year adjusted EHR^*^**  **(95% CI)** |
| --- | --- | --- | --- | --- | --- |
| **<30**^†^ | hrHPV- | 1 | 1.00 (1.00 to 1.00) | Ref | Ref |
|  | hrHPV+ | 19 | 0.89 (0.83 to 0.93) | - (-) | - (-) |
| **30-44** | hrHPV- | 21 | 0.84 (0.76 to 0.90) | Ref | Ref |
|  | hrHPV+ | 118 | 0.89 (0.87 to 0.91) | 0.64 (0.38 to 1.08) | 0.58 (0.34 to 0.98) |
| **45-59** | hrHPV- | 48 | 0.66 (0.56 to 0.73) | Ref | Ref |
|  | hrHPV+ | 189 | 0.75 (0.71 to 0.78) | 0.71 (0.50 to 1.00) | 0.66 (0.47 to 0.95) |
| **60-74** | hrHPV- | 95 | 0.47 (0.38 to 0.55) | Ref | Ref |
|  | hrHPV+ | 210 | 0.62 (0.57 to 0.67) | 0.57 (0.43 to 0.76) | 0.65 (0.49 to 0.87) |
| **>74** | hrHPV- | 144 | 0.29 (0.21 to 0.39) | Ref | Ref |
|  | hrHPV+ | 286 | 0.40 (0.33 to 0.47) | 0.57 (0.47 to 0.71) | 0.55 (0.42 to 0.71) |

^†^ No estimates for women under 30 due to insufficient outcome events.

^*^ EHRs were adjusted for age at cancer diagnosis as a spline term with 3 degrees of freedom, time since cancer diagnosis in 1-year bands, International Federation of Gynecology and Obstetrics (FIGO) stage, and education.
