# Supplementary material for: High-risk human papillomavirus status and prognosis in invasive cervical cancer: A nationwide cohort study
Source: PLoS Med. 2018 Oct 1;15(10):e1002666. doi: 10.1371/journal.pmed.1002666 (PMC6166926; doi:10.1371/journal.pmed.1002666)
Supplement: S2 Table — (DOCX) [file pmed.1002666.s002.docx]

**S2 Table. Five-year relative survival ratios (RSRs) and 5-year excess hazard ratios (EHRs) in relation to high-risk human papillomavirus (hrHPV) status, by FIGO stage.**

| **FIGO stage** | **hrHPV status** | **Deaths**  **(n=1131)** | **5-year RSR**  **(95% CI)** | **5-year crude EHR**  **(95% CI)** | **5-year adjusted EHR^*^**  **(95% CI)** |
| --- | --- | --- | --- | --- | --- |
| **IA** | hrHPV- | 6 | 1.00 (0.91 to 1.02) | Ref | Ref |
|  | hrHPV+ | 21 | 0.98 (0.96 to 0.99) | 2.63 (0.01 to 963.90) | 1.13 (0.16 to 8.20) |
| **IB** | hrHPV- | 67 | 0.77 (0.69 to 0.83) | Ref | Ref |
|  | hrHPV+ | 228 | 0.87 (0.84 to 0.89) | 0.53 (0.36 to 0.78) | 0.59 (0.40 to 0.86) |
| **II** | hrHPV- | 75 | 0.44 (0.34 to 0.54) | Ref | Ref |
|  | hrHPV+ | 240 | 0.64 (0.59 to 0.68) | 0.53 (0.39 to 0.73) | 0.59 (0.43 to 0.81) |
| **III+** | hrHPV- | 161 | 0.17 (0.12 to 0.24) | Ref | Ref |
|  | hrHPV+ | 334 | 0.28 (0.24 to 0.33) | 0.57 (0.44 to 0.70) | 0.62 (0.50 to 0.77) |

FIGO= International Federation of Gynecology and Obstetrics

^*^ EHRs were adjusted for age at cancer diagnosis as a spline term with 3 degrees of freedom, time since cancer diagnosis in 1-year bands, and education.
