# Supplementary material for: High-risk human papillomavirus status and prognosis in invasive cervical cancer: A nationwide cohort study
Source: PLoS Med. 2018 Oct 1;15(10):e1002666. doi: 10.1371/journal.pmed.1002666 (PMC6166926; doi:10.1371/journal.pmed.1002666)
Supplement: S3 Table — (DOCX) [file pmed.1002666.s003.docx]

**S3 Table. Characteristics of confirmed cases by availability of valid blocks.**

| **Characteristic** | **FFPE blocks retrieved (n=2850)**  **n (%)** | **FFPE blocks not retrieved (n=1404)**  **n (%)** | **Total (n=4254)**  **n (%)** | **P value** |
| --- | --- | --- | --- | --- |
| **Age at cancer diagnosis** |  |  |  |  |
| <30 | 167 (5.9) | 110 (7.8) | 277 (6.5) |  |
| 30-44 | 933 (32.7) | 465 (33.1) | 1398 (32.9) |  |
| 45-59 | 702 (24.6) | 319 (22.7) | 1021 (24.0) |  |
| 60-74 | 540 (18.9) | 269 (19.2) | 809 (19.0) |  |
| >74 | 508 (17.8) | 241 (17.2) | 749 (17.6) | 0.12 |
| **FIGO stage** |  |  |  |  |
| IA | 530 (18.6) | 332 (23.6) | 862 (20.3) |  |
| IB | 1157 (40.6) | 528 (37.6) | 1685 (39.6) |  |
| II | 577 (20.2) | 239 (17.0) | 816 (19.2) |  |
| III+ | 586 (20.6) | 305 (21.7) | 891 (20.9) | <0.001 |
| **Histological types** |  |  |  |  |
| Squamous cell carcinoma | 2111 (74.1) | 977 (69.6) | 3088 (72.6) |  |
| Adenocarcinoma | 528 (18.5) | 298 (21.2) | 826 (19.4) |  |
| Adenosquamous cell carcinoma | 119 (4.2) | 45 (3.2) | 164 (3.9) |  |
| Others rare carcinomas | 92 (3.2) | 84 (6.0) | 176 (4.1) | <0.001 |
| **Mode of detection** |  |  |  |  |
| Symptomatic cancer | 2042 (71.6) | 970 (69.1) | 3012 (70.8) |  |
| Screen-detected cancer | 808 (28.4) | 434 (30.9) | 1242 (29.2) | 0.08 |
| **Education** |  |  |  |  |
| Low | 816 (28.6) | 373 (26.6) | 1189 (28.0) |  |
| Middle | 1247 (43.8) | 623 (44.4) | 1870 (44.0) |  |
| High | 723 (25.4) | 385 (27.4) | 1108 (26.0) |  |
| Missing | 64 (2.2) | 23 (1.6) | 87 (2.0) | 0.19 |

FFPE, formalin-fixed paraffin-embedded.

FIGO, International Federation of Gynecology and Obstetrics.
