# Supplementary material for: High-risk human papillomavirus status and prognosis in invasive cervical cancer: A nationwide cohort study
Source: PLoS Med. 2018 Oct 1;15(10):e1002666. doi: 10.1371/journal.pmed.1002666 (PMC6166926; doi:10.1371/journal.pmed.1002666)
Supplement: S4 Table — (DOCX) [file pmed.1002666.s004.docx]

**S4 Table. One-year, 3-year, 5-year and 10-year excess hazard ratios (EHRs) in relation to high-risk human papillomavirus (hrHPV) status.**

| **hrHPV status** | **Cases**  **(n=2845)** | **Deaths**  **(n=1131)** |  | **1-year EHR**  **(95% CI)** | **3-year EHR**  **(95% CI)** | **5-year EHR**  **(95% CI)** | **10-year EHR**  **(95% CI)** |
| --- | --- | --- | --- | --- | --- | --- | --- |
| hrHPV- | 552 | 309 |  | Ref | Ref | Ref | Ref |
| hrHPV+ | 2293 | 822 | Crude | 0.30 (0.24 to 0.38) | 0.38 (0.31 to 0.45) | 0.45 (0.38 to 0.52) | 0.47 (0.41 to 0.55) |
|  |  |  | Adjusted* | 0.49 (0.39 to 0.61) | 0.54 (0.45 to 0.65) | 0.61 (0.52 to 0.71) | 0.64 (0.55 to 0.75) |

^*^ EHRs were adjusted for age at cancer diagnosis as a spline term with 5 degrees of freedom, time since cancer diagnosis in 1-year bands, International Federation of Gynecology and Obstetrics (FIGO) stage, and education.
