# Supplementary material for: High-risk human papillomavirus status and prognosis in invasive cervical cancer: A nationwide cohort study
Source: PLoS Med. 2018 Oct 1;15(10):e1002666. doi: 10.1371/journal.pmed.1002666 (PMC6166926; doi:10.1371/journal.pmed.1002666)
Supplement: S5 Table — (DOCX) [file pmed.1002666.s005.docx]

## S5 Table. Five-year relative survival ratios (RSRs) and 5-year excess hazard ratios (EHRs) in relation to high-risk human papillomavirus (hrHPV) status, by mode of detection.

| **Mode of detection** | **hrHPV status** | **Death**  **(n=1131)** | **5-year RSR**  **(95% CI)** | **5-year adjusted EHR^*^**  **(95% CI)** |
| --- | --- | --- | --- | --- |
| Symptomatic cancer | hrHPV- | 295 | 0.45 (0.40 to 0.50) | Ref |
| Symptomatic cancer | hrHPV+ | 784 | 0.64 (0.61 to 0.66) | 0.63 (0.53 to 0.74) |
| Screen-detected cancer | hrHPV- | 14 | 0.93 (0.86 to 0.97) | Ref |
| Screen-detected cancer | hrHPV+ | 38 | 0.98 (0.96 to 0.99) | 0.19 (0.06 to 0.60) |

^*^ EHRs were adjusted for age at cancer diagnosis as a spline term with 5 degrees of freedom, time since cancer diagnosis in 1-year bands, International Federation of Gynecology and Obstetrics (FIGO) stage and education.
