# Supplementary material for: High-risk human papillomavirus status and prognosis in invasive cervical cancer: A nationwide cohort study
Source: PLoS Med. 2018 Oct 1;15(10):e1002666. doi: 10.1371/journal.pmed.1002666 (PMC6166926; doi:10.1371/journal.pmed.1002666)
Supplement: S6 Table — (DOCX) [file pmed.1002666.s006.docx]

**S6 Table. Five-year relative survival ratios (RSRs) and 5-year excess hazard ratios (EHRs) in relation to high-risk human papillomavirus (hrHPV) status, in high-grade tumors.**

| **hrHPV status** | **Cases**  **(n=221)** | **Deaths**  **(n=125)** | **5-year RSR**  **(95% CI)** |  | **5-year EHR (95% CI)** | | |
| --- | --- | --- | --- | --- | --- | --- | --- |
|  |  |  |  |  | **Crude** |  | **Adjusted^*^** |
| hrHPV- | 64 | 44 | 0.40 (0.27 to 0.52) |  | Ref |  | Ref |
| hrHPV+ | 157 | 81 | 0.56 (0.47 to 0.64) |  | 0.54 (0.36 to 0.82) |  | 0.44 (0.29 to 0.68) |

^*^EHRs were adjusted for age at cancer diagnosis as a spline term with 5 degrees of freedom, time since cancer diagnosis in 1-year bands, International Federation of Gynecology and Obstetrics (FIGO) stage, and education.
