# Supplementary material for: High-risk human papillomavirus status and prognosis in invasive cervical cancer: A nationwide cohort study
Source: PLoS Med. 2018 Oct 1;15(10):e1002666. doi: 10.1371/journal.pmed.1002666 (PMC6166926; doi:10.1371/journal.pmed.1002666)
Supplement: S7 Table — (DOCX) [file pmed.1002666.s007.docx]

# Sensitivity analysis using both Luminex and PCR HPV16-E7 and HPV18-E6 results.

**S7 Table. Five-year relative survival ratios (RSRs) and 5-year excess hazard ratios (EHRs) in relation to high-risk human papillomavirus (hrHPV) status based on L1 region and HPV16-E7/HPV18-E6.**

| **hrHPV status** | **Cases**  **(n=2845)** | **Deaths**  **(n=1131)** | **5-year RSR**  **(95% CI)** |  | **5-year EHR (95% CI)** | | |
| --- | --- | --- | --- | --- | --- | --- | --- |
|  |  |  |  |  | **Crude** |  | **Adjusted^*^** |
| hrHPV- | 463 | 270 | 0.53 (0.48 to 0.58) |  | Ref |  | Ref |
| hrHPV+ | 2382 | 861 | 0.74 (0.72 to 0.76) |  | 0.43 (0.37 to 0.51) |  | 0.63 (0.53 to 0.75) |

^*^ EHRs were adjusted for age at cancer diagnosis as a spline term with 5 degrees of freedom, time since cancer diagnosis in 1-year bands, International Federation of Gynecology and Obstetrics (FIGO) stage, and education.
