# Supplementary material for: High-risk human papillomavirus status and prognosis in invasive cervical cancer: A nationwide cohort study
Source: PLoS Med. 2018 Oct 1;15(10):e1002666. doi: 10.1371/journal.pmed.1002666 (PMC6166926; doi:10.1371/journal.pmed.1002666)
Supplement: S8 Table — (DOCX) [file pmed.1002666.s008.docx]

# Sensitivity analysis using both Luminex and PCR HPV16-E7 and HPV18-E6 results.

**S8 Table. Five-year relative survival ratios (RSRs) and 5-year excess hazard ratios (EHRs) in relation to high-risk human papillomavirus (hrHPV) status based on L1 region and HPV16-E7/HPV18-E6, by age at cancer diagnosis.**

| **Age at cancer diagnosis** | **hrHPV status** | **Deaths**  **(n=1131)** | **5-year RSR**  **(95% CI)** | **5-year crude EHR**  **(95% CI)** | **5-year adjusted EHR^*^**  **(95% CI)** |
| --- | --- | --- | --- | --- | --- |
| **<30**^†^ | hrHPV- | 0 | 1.00 (1.00 to 1.00) | Ref | Ref |
|  | hrHPV+ | 20 | 0.89 (0.83 to 0.93) | - (-) | - (-) |
| **30-44** | hrHPV- | 18 | 0.80 (0.69 to 0.87) | Ref | Ref |
|  | hrHPV+ | 121 | 0.89 (0.87 to 0.91) | 0.47 (0.28 to 0.80) | 0.45 (0.27 to 0.77) |
| **45-59** | hrHPV- | 43 | 0.65 (0.55 to 0.73) | Ref | Ref |
|  | hrHPV+ | 194 | 0.75 (0.71 to 0.78) | 0.70 (0.48 to 1.00) | 0.66 (0.45 to 0.95) |
| **60-74** | hrHPV- | 82 | 0.48 (0.39 to 0.57) | Ref | Ref |
|  | hrHPV+ | 223 | 0.61 (0.56 to 0.66) | 0.59 (0.44 to 0.79) | 0.66 (0.49 to 0.89) |
| **>74** | hrHPV- | 127 | 0.30 (0.21 to 0.40) | Ref | Ref |
|  | hrHPV+ | 303 | 0.39 (0.33 to 0.46) | 0.64 (0.49 to 0.83) | 0.65 (0.50 to 0.85) |

^†^ No estimates for women under 30 due to insufficient outcome events.

^*^ EHRs were adjusted for age at cancer diagnosis as a spline term with 3 degrees of freedom, time since cancer diagnosis in 1-year bands, International Federation of Gynecology and Obstetrics (FIGO) stage, and education.
