# Supplementary material for: High-risk human papillomavirus status and prognosis in invasive cervical cancer: A nationwide cohort study
Source: PLoS Med. 2018 Oct 1;15(10):e1002666. doi: 10.1371/journal.pmed.1002666 (PMC6166926; doi:10.1371/journal.pmed.1002666)
Supplement: S9 Table — (DOCX) [file pmed.1002666.s009.docx]

# Sensitivity analysis using both Luminex and PCR HPV16-E7 and HPV18-E6 results.

**S9 Table. Five-year relative survival ratios (RSRs) and 5-year excess hazard ratios (EHRs) in relation to high-risk human papillomavirus (hrHPV) status based on L1 region and HPV16-E7/HPV18-E6, by FIGO stage.**

| **FIGO stage** | **hrHPV status** | **Deaths**  **(n=1131)** | **5-year RSR**  **(95% CI)** | **5-year crude EHR**  **(95% CI)** | **5-year adjusted EHR^*^**  **(95% CI)** |
| --- | --- | --- | --- | --- | --- |
| **IA** | hrHPV- | 6 | 0.98 (0.88 to 1.01) | Ref | Ref |
|  | hrHPV+ | 21 | 0.98 (0.96 to 0.99) | 0.50 (0.06 to 4.06) | 0.95 (0.14 to 6.49) |
| **IB** | hrHPV- | 57 | 0.75 (0.66 to 0.82) | Ref | Ref |
|  | hrHPV+ | 238 | 0.87 (0.84 to 0.89) | 0.48 (0.32 to 0.73) | 0.54 (0.36 to 0.81) |
| **II** | hrHPV- | 65 | 0.42 (0.31 to 0.53) | Ref | Ref |
|  | hrHPV+ | 250 | 0.63 (0.58 to 0.68) | 0.50 (0.36 to 0.70) | 0.57 (0.40 to 0.79) |
| **III+** | hrHPV- | 142 | 0.18 (0.12 to 0.25) | Ref | Ref |
|  | hrHPV+ | 353 | 0.27 (0.23 to 0.32) | 0.63 (0.51 to 0.78) | 0.69 (0.56 to 0.86) |

FIGO = International Federation of Gynecology and Obstetrics.

^*^ EHRs were adjusted for age at cancer diagnosis as a spline term with 5 degrees of freedom, time since cancer diagnosis in 1-year bands, and education.
