# Supplementary material for: High-risk human papillomavirus status and prognosis in invasive cervical cancer: A nationwide cohort study
Source: PLoS Med. 2018 Oct 1;15(10):e1002666. doi: 10.1371/journal.pmed.1002666 (PMC6166926; doi:10.1371/journal.pmed.1002666)
Supplement: S10 Table — (DOCX) [file pmed.1002666.s010.docx]

## S10 Table. Tumor human papillomavirus (HPV) status and pre-diagnostic HPV-testing results.

| **Tumor HPV status (based on L1 region)** | **HPV-testing results** | | **Total** |
| --- | --- | --- | --- |
|  | **hrHPV-negative**  **n (%)** | **hrHPV-positive**  **n (%)** | **n (%)** |
| HPV-negative | 1 (25%)^a^ | 3 (75%) | 4 (100%) |
| HPV-positive | 1 (2%)^b^ | 50 (98%) | 51 (100%) |
| Total | 2 (4.6%) | 53 (96.4%) | 55 (100%) |

hrHPV, high-risk human papillomavirus.

^a^ Adenocarcinoma diagnosed at stage II, and the screening test was performed in year 2007 and the cancer diagnosis was in year 2009.

^b^ HPV-positive for type HPV73 in cervical tumor.
