# Supplementary material for: High-risk human papillomavirus status and prognosis in invasive cervical cancer: A nationwide cohort study
Source: PLoS Med. 2018 Oct 1;15(10):e1002666. doi: 10.1371/journal.pmed.1002666 (PMC6166926; doi:10.1371/journal.pmed.1002666)
Supplement: S1 Text — (PDF) [file pmed.1002666.s011.pdf]

High-risk human papillomavirus status and invasive cervical cancer prognosis

Analysis protocol

PhD student: Jiayao Lei

Department of Medical Epidemiology and Biostatistics, Karolinska Institutet

Stockholm, Sweden

Analysis_protocol_

** **High-risk human papillomavirus status and invasive cervical cancer prognosis** **

Created: 2016-01-14 by Jiayao Lei

1 OBJECTIVE AND HYPOTHESES

Aim: to evaluate the association between tumor HPV status and invasive cervical cancer (ICC) prognosis.

Hypothesis: women with high-risk human papillomavirus (hrHPV) positive tumors have better prognosis than women with hrHPV-negative tumors.

2 STUDY POPULATION

All women diagnosed with ICC in Sweden between 2002 and 2011, as retrieved from the National Cancer Registry.

Diagnostic formalin-fixed paraffin-embedded (FFPE) samples has been collected from different laboratories across Sweden and reviewed by a senior gynecologist as to ICC diagnosis.

Exclusion criteria:

- medical charts review indicating disease other than primary ICC;
- no available FFPE samples for HPV genotyping;
- unsatisfactory material for representative HPV genotyping, as determined by senior pathologist review of HPV-negative diagnostic FFPE samples.

3 MEASUREMENTS AND VARIABLES

3.1 Outcome variable

All causes of death in case women, collected from the Total Population Registry

1=yes/death, 0=no/alive.

3.2 Exposure and covariates

Main exposure: tumor hrHPV status (hrHPV+ vs. hrHPV-)

- **HPV genotyping:** L1 region
  - → 13 high-risk types (16, 18, 31, 33, 35, 39, 45, 51, 52, 56, 58, 59, 68)
  - → HPV types not established as oncogenic (low-risk types) (HPV 6, 11, 26, 30, 40, 42, 43, 53, 54, 61, 66, 67, 69, 70, 73, 74, 81, 82, 83, 86, 87, 89, 90, 91)

Other covariates:

- Age at cancer diagnosis (<30, 30-44, 45-59, 60-74 and >74)
- FIGO stage (IA, IB, II and III+)
- Education (low, middle, high)
- Treatment (conization/radical trachelectomy, surgical treatment (hysterectomy), surgical treatment and radiation/chemotherapy, radiation and/or chemotherapy, palliation)
- Mode of detection (screen-detected, symptomatic cancer)
- Histological types (squamous cell carcinoma, adenocarcinoma, adenosquamous cell carcinoma and other rare histological types)

4 STATISTICAL ANALYSES

Kaplan-Meier curves by hrHPV status to inspect difference in mortality over time

Mortality rate by hrHPV status

Cox regression: hazard ratios with 95% confidence intervals (CIs)

Updated_Analysis_protocol_

** **High-risk human papillomavirus status and invasive cervical cancer prognosis** **

Updated: 2017-03-30 by Jiayao Lei (changes were marked as *Italic*)

Updated: 2017-10-20 by Jiayao Lei (changes were marked as *Italic*)

1 OBJECTIVE AND HYPOTHESES

Aim: to evaluate the association between tumor HPV status and invasive cervical cancer (ICC) prognosis.

Hypothesis: women with high-risk human papillomavirus (hrHPV) positive tumors have better prognosis than women with hrHPV-negative tumors.

2 STUDY POPULATION

All women diagnosed with ICC in Sweden between 2002 and 2011, as retrieved from the National Cancer Registry.

Diagnostic formalin-fixed paraffin-embedded (FFPE) samples has been collected from different laboratories across Sweden and reviewed by a senior gynecologist as to ICC diagnosis.

Exclusion criteria:

- medical charts review indicating disease other than primary ICC, including:
    - not primary cervical origin
    - not epithelial
    - recurrence
- no available FFPE samples for HPV genotyping;
- unsatisfactory material for representative HPV genotyping, as determined by senior pathologist review of HPV-negative diagnostic FFPE samples.

3 MEASUREMENTS AND VARIABLES

3.1 Outcome variable

All causes of death in case women, collected from the Total Population Registry

1=yes/death, 0=no/alive.

3.2 Exposure and covariates

Main exposure: tumor hrHPV status (hrHPV+ vs. hrHPV-)

- **<u>HPV genotyping</u>:** L1 region
  - → 13 high-risk types (16, 18, 31, 33, 35, 39, 45, 51, 52, 56, 58, 59, 68)
  - → HPV types not established as oncogenic (low-risk types) (HPV 6, 11, 26, 30, 40, 42, 43, 53, 54, 61, 66, 67, 69, 70, 73, 74, 81, 82, 83, 86, 87, 89, 90, 91)

Other covariates:

- Age at cancer diagnosis (<30, 30-44, 45-59, 60-74 and >74)
  - include as both adjustment and stratification in analysis
- FIGO stage (IA, IB, II and III+)
  - include as both adjustment and stratification in analysis
- Education (low, middle, high)
- *Treatment (conization/radical trachelectomy, surgical treatment (hysterectomy), surgical treatment and radiation/chemotherapy, radiation and/or chemotherapy, palliation)*
  - *removed in this analysis since strongly related to FIGO stage*
- Mode of detection (screen-detected; symptomatic cancer)
  - Stratification

- Histological types (squamous cell carcinoma; adenocarcinoma, adenosquamous cell carcinoma and other rare histological types)
    - Stratification

4 STATISTICAL ANALYSES

Relative survival added as main outcome measure.

Poisson regression model instead of Cox regression model chosen.

Relative survival ratios (RSRs) and excess hazard ratios (EHRs) in relation to the general female population with comparable age and calendar period were estimated with 95% CI.

Note: Slight non-proportionality detected, but driven mostly by the under 45, followed by stage (very good proportionality overall, except for the IAs) and hrHPV status (mild time-varying effects). Decision to use Poisson regression model with spline term for age adjustment to better take care of the non-proportionality from age at cancer diagnosis and estimate relative survival.

5 SENSITIVITY OR OTHER RELATED ANALYSES

5.1 PCR HPV16-E7 and HPV 18-E6 testing for Luminex HPV-negative cases.

Since loss of the L1 region could occur in tumors, whereas the oncogenic E7 and E6 regions are more likely to be retained. HPV16-E7 and HPV18-E6 real-time (RT) PCR results will be performed on all L1-negative samples, and the results included as sensitivity analysis in same manner as analysis based on Luminex results.

5.2 Representativeness of the diagnostic blocks for cancer cases 2002-2011

Compare the availablity of FFPE blocks in all biobanks in Sweden to ensure the samples.

5.3 Comparison of cases by availability of valid blocks regarding covariates

Similar to table 1 including all covariates, to ensure the samples included are representative for the whole nation's load of ICC cases 2002-2011

5.4 Comparison of HPV status across calender year

A frequency table. To examine/exclude the possibility that longer storage time of FFPE blocks might result in lower detectability of HPV.

5.5 A subgroup analysis in tumors classified as high grade (non-prespecified analysis)

Based on comments from an audient in EUROGIN 2017, a subgroup analysis would be appropriate, to be added to examine the hrHPV status and prognosis by tumor grade. Thus hrHPV status and association with prognosis in all tumors noted as high-grade by chart/histopathological review will be considered separately.
